# ED_50_ of remimazolam combined with different doses butorphanol for first trimester artificial abortion

**DOI:** 10.3389/fmed.2024.1385998

**Published:** 2024-04-18

**Authors:** Jinming Chen, Xiaoling Li, Zilan Hu, Yuling Zheng, Ying Mai, Zhongqi Zhang

**Affiliations:** ^1^Department of Anesthesiology, The Affiliated Shunde Hospital of Jinan University, Foshan, China; ^2^Department of Gynecology, The Affiliated Shunde Hospital of Jinan University, Foshan, China

**Keywords:** remimazolam, butorphanol, effective dose, painless abortion, up-and-down method

## Abstract

**Introduction:**

Remimazolam (RMZ) is a novel intravenous sedative drug of ultra-short benzodiazepine. The optimal dose of RMZ plus butorphanol for sedation during first trimester artificial abortion is unknown. Therefore, the present study aimed to evaluate the median effective dose (ED_50_) of RMZ combined with different doses of butorphanol on the sedative effect for first-trimester artificial abortion.

**Methods:**

Sixty-one female patients were randomly assigned to Group B10 (31 patients) and Group B15 (30 patients). RMZ was administered 5 min after IV butorphanol at doses of 10 μg/kg (Group B10) and 15 μg/kg (Group B15). Cervical dilatation at the time of using a cervical dilating rod, if the patient has body movement and affects the gynecologist’s operation, we define it as “Ineffective.” Therefore, the dose of RMZ was increased in the next patient. Otherwise, it was defined as “Effective,” and the dose of RMZ was reduced in the next patient. According to the pre-experiment, the first dose of RMZ in the first patient was 0.35 mg/kg, and the adjacent geometric dose ratio was 0.9. The centered isotonic regression was performed to determine the ED_50_ of RMZ. The total RMZ dose administered, recovery time, and anesthesia-related adverse events were all recorded.

**Results:**

The ED_50_ (90% CI) of RMZ was 0.263 (0.215–0.310) mg/kg in Group B10, and 0.224 (0.191–0.261) mg/kg in Group B15, respectively. The recovery time in Group B10 was significantly shorter than in Group B15 (9.8 ± 2.3 vs. 12.5 ± 3.6 min, *p* ≤ 0.001). There was no significant difference in the incidence rate of all anesthesia-related adverse events between the two groups (*p* > 0.05).

**Conclusion:**

The ED50 of RMZ combined with a 10 μg/kg or 15 μg/kg dose of butorphanol was 0.263 and 0.224 mg/kg during painless first trimester artificial abortion. However, RMZ combined with a 10 μg/kg dose of butorphanol seems to have a shorter recovery time.

**Clinical trial registration:**

https://www.chictr.org.cn/bin/project/edit?pid=166623.

## 1 Introduction

Artificial abortion is one of the most widely accepted methods of contraceptive failure among all early abortions (1). Artificial abortion is usually a relatively short operation, which can be completed within 3–5 min. However, pulling and dilating the cervical canal and sucking and scraping the uterine wall will cause severe pain. Many patients will experience involuntary limb movements that significantly increase the risk of surgical abortion (2). Therefore, painless abortion frequently necessitates general anesthesia to alleviate the patient’s physical discomfort during the procedure.

Butorphanol is a mixture of opioid receptor agonists and antagonists that can produce analgesic effects through kappa receptors, making it particularly suitable for treating visceral pain. Butorphanol has recently been widely used in outpatient surgery due to its good sedative and analgesic effects with a lower degree of respiratory depression compared to traditional potent opioid drugs (sufentanil or fentanyl) (3). In addition, it can also effectively alleviate remifentanil-induced hyperalgesia (4, 5). However, the sedative effect of butorphanol can result in side effects such as post-operative drowsiness and dizziness (6).

Remimazolam (RMZ) is an ultra-short acting benzodiazepine with the rapid induction of sedation, fast recovery, and no injection-site pain (7). These characteristics make RMZ especially suitable for procedures such as gastroenteroscopy and hysteroscopy (8, 9). Furthermore, a recent study revealed that RMZ pre-trials reduced the frequency and intensity of injection pain caused by propofol in abortion (10). However, it is recommended to be used in combination with opioids for optimal effectiveness during procedural sedation (11).

We will use RMZ in combination with different doses of butorphanol in painless artificial abortion to determine the efficacy of the RMZ. The optimal dose of RMZ plus butorphanol for sedation during a painless abortion is unknown. There is no relevant research exists. Therefore, the effects of different doses of butorphanol on the median effective dose (ED_50_) of the RMZ in inhibiting the response of cervical dilatation were investigated to provide a reference for the safety and rational use of the drug in painless artificial abortion.

## 2 Materials and methods

### 2.1 Study design

The present study is a prospective, randomized, and double-blind study. Ethics Committee of of the Affiliated Shunde Hospital of Jinan University approved the study (number: JDSY-LL-2022004, 10/04/2022). The trial was registered at the Chinese Clinical Trial Registry (www.chictr.org.cn, number: ChiCTR2200059793, 11/05/2022). All trial procedures were performed in accordance with the relevant guidelines and regulations set by the Affiliated Shunde Hospital of Jinan University.

### 2.2 Participants

All patients who had an artificial abortion from May to September 2022 were included in the study. Each patient was asked to sign an informed consent.

Inclusion criteria: perform elective artificial abortion; American Society of Anesthesiologists (ASA) I or II; clinically confirmed early-pregnancy by color doppler ultrasound (<12 weeks); between 18 and 49 years of age; body mass index (BMI) between 18 to 30 kg/m^2^; and without disease related to the cervix and no prior cervical surgery. Exclusion criteria: Refuse to participate; ASA class III or higher; allergy to RMZ or butorphanol; severe liver/kidney/cardiopulmonary/central nervous system dysfunction; a procedure time >10 min; and long-term use of sedative or analgesic medications.

### 2.3 Grouping and anesthesia management

In the present study, 61 patients were randomly assigned into one of the two groups: Group B10 (31 patients) and Group B15 (30 patients). All patients were given RMZ after 5 min of an intravenous (IV) administration of butorphanol 10 μg/kg (Group B10) or 15 μg/kg (Group B15).

Patients have fasted for more than 8 h, and drinking was prohibited for at least 2 h. After entering the operating room, venous access was obtained at the dorsum of the left hand using an IV infusion needle with a diameter of 0.6 mm, and oxygen was given through nasal straw at the flow rate of 3 L/min. Electrocardiogram (ECG), blood pressure (BP), and blood oxygen saturation (SpO_2_) were measured. The same experienced gynecologist and anesthesiologist performed all surgical procedures and anesthesia. The patient received IV butorphanol (Jiangsu Hengrui Medicine Co., China, diluted to 10 mL with normal saline, lot number: 220129BP), 10 μg/kg (Group B10) or 15 μg/kg (Group B15) at least 5 min before IV administration of RMZ (Jiangsu Hengrui Medicine Co., China, diluted to 1 mg/mL with normal saline, lot number: 220326AK) for sedation. The maximal consumption of butorphanol was 1 mg in both groups.

The most painful part of the procedure was reported to be cervical dilation (12). Cervical dilatation at the time of using a cervical dilating rod, if the patient has body movement and affects the gynecologist’s operation, we define it as “Ineffective.” Therefore, the next patient received an increased RMZ dose. Otherwise, it was defined as “Effective,” and the RMZ dose was reduced in the next patient. According to the pre-experiment, the first RMZ dose in the first patient was 0.35 mg/kg, and the adjacent geometric dose ratio was 0.9. Therefore, the doses for the A and B groups were as follows: 0.35, 0.315, 0.283, 0.255, 0.229, 0.206, 0.186, and 0.167 mg/kg. If “Ineffective” occurs, intravenous injection of RMZ at a dose of 0.05 mg/kg can be administered to deepen anesthesia, which can be repeated. According to the instructions, the number of RMZ injections within 15 min should not exceed 5 times. If repeated injections of RMZ still fail to achieve the required depth of anesthesia, propofol should be added, and the patient should be excluded from the trial.

All patients were transported to the Post Anesthesia Care Unit (PACU) after the surgery until they awoke. The ECG, BP, and SpO_2_ were also continuously monitored every 5 min for at least 30 min. Ability to walk independently, stable vital signs and no obvious adverse reactions were the criteria for transferring out of the PACU.

### 2.4 Outcome assessments

The primary outcome measure: the dose of RMZ for each patient was measured using the up-and-down method.

The secondary outcome: SBP/DBP, heart rate (HR), and SpO_2_ were recorded at 5 min after entering the operating room (T1) and immediately after IV injection of RMZ (T2). Respiratory depression (SpO_2_ < 90%), hypotension, bradycardia, injection-site pain, uterine contraction pain, dizziness, and post-operative nausea and vomiting (PONV) were also measured. In addition, the initial RMZ dose, the total RMZ dose, the total duration of the surgical procedure, and the recovery time were all recorded.

It was defined as injection-site pain when the patient frowned or complained of pain in the back of the hand or the ipsilateral arm escape reflex. The recovery time was the duration between the last RMZ injection and the eye-opening on command. Adverse events were managed as follows: hypotension (20% reduction in MAP compared to baseline) IV ephedrine 6–12 mg; bradycardia (HR < 50 beats/min) IV atropine 0.25–1 mg; respiratory depression (SpO_2_ < 90%) maintain ventilation with a mask or laryngeal mask; PONV IV tropisetron 2 mg; and uterine contraction pain (VAS score ≥ 4) IV sufentanil 3–5 μg.

### 2.5 Blinding method

The randomization assignments were computer generated and then group information was sealed in an opaque envelope. All surgical procedures and anesthesia were performed by the same experienced gynecologist and anesthesiologist. An independent observer who was also blinded to group assignment and recorded the patients’ vital signs and any anesthesia-related adverse events. The butorphanol was diluted with normal saline to 10 mL which appeared colorless and odorless, the 10 mL transparent syringe without any label was placed in a tray together with RMZ for the recruited patient, an independent researcher was responsible for drug distribution. Both the anesthesiologist and data recorder were blinded to the drug being injected.

### 2.6 Statistical analysis

The sample size calculation was determined using Dixon’s up-and-down method (13). For statistical analysis, seven crossovers (Effective to Ineffective) are required. We performed statistical analyses using SPSS version 20.0 (Inc., Chicago, IL, United States). Data were presented as means ± standard deviations (SD), median [range], or *n* (%), depending on the distribution of the data. Normally distributed continuous variables were compared using t-test, while the Mann–Whitney *U* test was used for non-normally distributed continuous variables. Categorical variables were compared using the chi-square or Fisher exact probability test in two groups. The centered isotonic regression of R Language was performed to determine the ED_50_ (14). *p* < 0.05 was indicated to represent statistically significantly difference.

## 3 Results

### 3.1 Patients information

**Figure 1:**
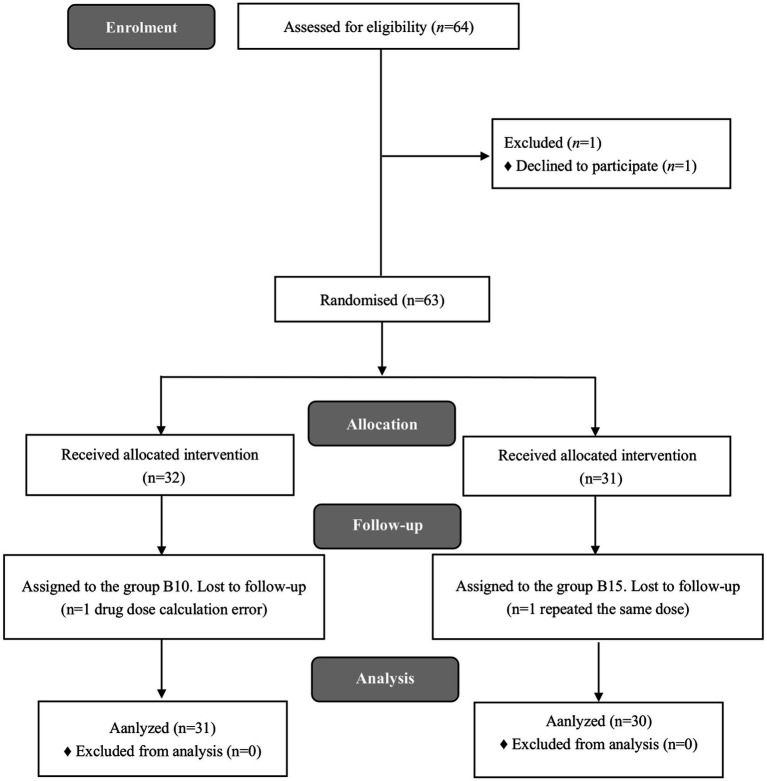
Flow diagram of the study.

A total of 64 female participants were enrolled in the present study. Three participants were excluded, and 61participants completed the study successfully. The flow diagram of the study is shown in Figure 1. Table 1 demonstrates patients’ characteristic data for all patients. There were no statistically significant differences (*p* > 0.05) between the 2 groups in terms of ASA, age, height, weight, BMI, gestational week, number of times pregnant, number of cesarean sections, number of vaginal deliveries, and number of abortions.

**Table 1:** Patients’ characteristics.

Characteristics	Group B10, *n* = 31	Group B15, *n* = 30	*p*-value
ASA (I/II)	30/1	29/1	—
Age (years)	29.8 ± 7.5	30.0 ± 6.3	0.870
Height (cm)	158.6 ± 1.2	159.3 ± 1.0	0.597
Weight (kg)	55.3 ± 7.1	54.6 ± 6.7	0.712
BMI (kg/m^2^)	22.0 ± 2.4	21.5 ± 2.2	0.410
Gestational week	6.1 ± 0.9	6.3 ± 0.9	0.758
Number of times pregnant	3 [2–4]	3 [3–4]	0.133
Number of cesarean sections	0 [0–0]	0 [0–0]	0.754
Number of vaginal deliveries	1 [0–2]	2 [1–2]	0.222
Number of abortions	0 [0–1]	0 [0–1]	0.513

Data are expressed as means ± standard deviations (SD) or median [range]. Group B10 received butorphanol 10 μg/kg. Group B15 received butorphanol 15 μg/kg.

### 3.2 ED_50_ of RMZ

**Figure 2:**
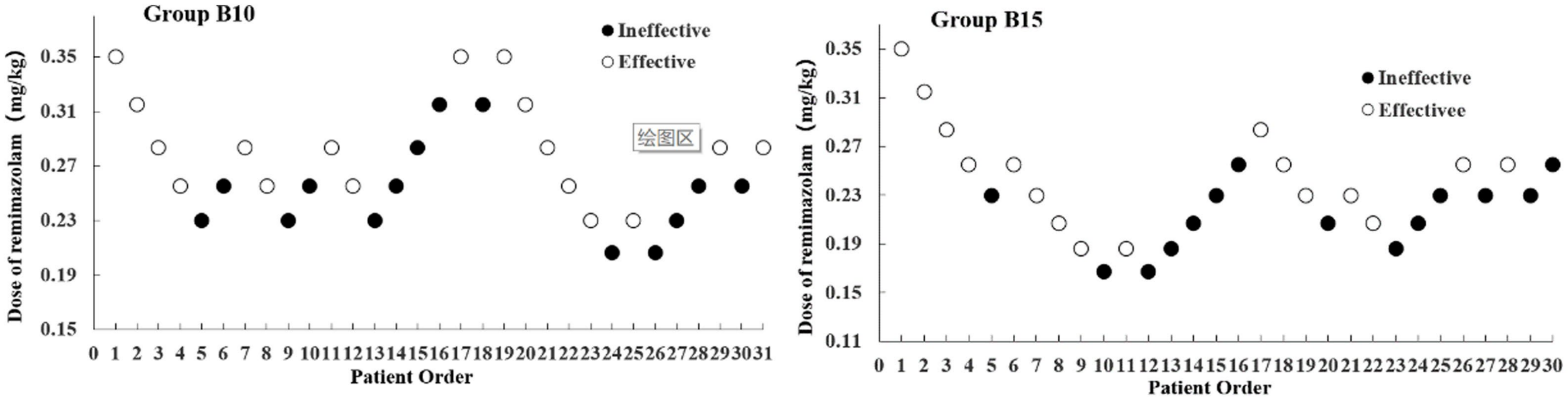
Dixon’s up-down method plots for two groups. The white and black dots represent the “Effective” and “Ineffective” patient order, respectively.

The sample size was achieved after seven effective/ineffective crossovers using the up-and-down method (Figure 2). There were 31 and 30 patients in Groups B10 and B15, respectively. Furthermore, 14 patients were ineffective and given RMZ as rescue therapy in both groups. The ED_50_ (90% CI) of the RMZ was 0.263 (0.215–0.310) mg/kg in Group B10, and 0.224 (0.191–0.261) mg/kg in Group B15, respectively (Table 2).

**Table 2:** Comparison of perioperative outcomes between the 2 groups.

Items	Group B10	Group B15	*p*-value
Initial dose of RMZ (mg)	14.9 ± 2.5	12.7 ± 2.7	0.002
Total dose of RMZ (mg)	17.9 ± 3.3	16.0 ± 3.7	0.037
Duration of procedure (min)	3.7 ± 1.0	3.5 ± 0.8	0.365
Recovery time (min)	9.8 ± 2.3	12.5 ± 3.6	0.001

Data are expressed as mean ± SD. The total dose of RMZ: initial dose plus additional dose. Students t-test was used to assess differences.

### 3.3 Perioperative outcomes

Table 3 displays the perioperative outcomes. The initial and total dosage of RMZ consumed in Group B10 was significantly higher than that in Group B15 (14.9 ± 2.5 vs. 12.7 ± 2.7 mg, 17.9 ± 3.3 vs. 16.0 ± 3.7 mg, *p* < 0.05, respectively). The procedure duration (3.7 ± 1.0 vs. 3.5 ± 0.8 min, *p* = 0.365) was not significantly different between the two groups. The recovery time in Group B10 was faster than in Group B15 (9.8 ± 2.3 vs. 12.5 ± 3.6 min, *p* < 0.001).

**Table 3:** ED_50_ of RMZ for two groups.

Group	ED_50_ (90% CI), mg/kg
Group B10	0.263 (0.215–0.310)
Group B15	0.224 (0.191–0.261)

The centered isotonic regression was used to determine ED_50_ (90% CI) of RMZ.

### 3.4 Hemodynamic changes and adverse events

The results that the reduction of MAP in both groups was about only 11%. HR, MAP, and SpO_2_ levels in the two groups at different time points have no statistical difference (*p* > 0.05) (Table 4). The incidence of PONV in the B10 and B15 groups was 3.2 and 16.7%, respectively. But there were no statistically differences between the 2 groups in the rate of all anesthesia-related adverse events (*p* > 0.05) (Table 5).

**Table 4:** Comparison of HR, MAP, and SpO_2_ of the two groups at different time points.

Parameters	Group	Time point		*p*-value
		T1	T2	
HR (beats/min)	Group B10	80.1 ± 14.7	83.1 ± 11.3	0.602
	Group B15	79.0 ± 12.6	81.1 ± 12.0	0.507
MAP (mmHg)	Group B10	87.8 ± 8.4	77.9 ± 9.4	0.196
	Group B15	86.4 ± 7.5	77.0 ± 6.4	0.660
SpO_2_ (%)	Group B10	99.5 ± 0.6	98.9 ± 1.2	0.596
	Group B15	99.5 ± 0.6	99.0 ± 0.9	0.720

Date are expressed as mean ± SD. T1: 5 min after entering the operating room; T2: Immediately after IV injection of RMZ. Students *t*-test was used to assess differences.

**Table 5:** Anesthesia-related adverse events.

Adverse events	Group B10 *n* = 31	Group B15 *n* = 30	*p*-value
SpO_2_ < 90%	0 (0)	0 (0)	—
Hypotension	2 (6.4)	2 (6.6)	—
Bradycardia	0 (0)	0 (0)	—
Injection-site pain	1 (3.2)	0 (0)	—
Injection-site pain VAS score	1 [0–0]	0 [0–0]	—
Uterine contraction pain	4 (12.9)	5 (16.7)	0.731
Uterine contraction pain VAS score	0 [0–0]	0 [0–0]	—
Dizziness	2 (6.5)	2 (6.7)	—
PONV	1 (3.2)	5 (16.7)	0.104

Data are expressed as *n* [%] or median [range]. A chi-square or Fisher exact probability test was used to assess differences of adverse events.

## 4 Discussion

Cervical dilatation during a painless artificial abortion can result in intense stimulation (12). Light or deep anesthesia can cause severe adverse events, posing many challenges for anesthesiologists regarding actual drug selection. The most common drug combination for painless artificial abortion is sedative and analgesic drugs. Due to the short duration of abortion surgery, anesthesiologists required rapid induction while also ensuring the safety and quality of the anesthesia. The minimum effective dose can achieve adequate anesthesia while reducing drug dosage and the incidence of adverse events.

Among the many methods for determining ED_50_, up-and-down method is quick and simple, and it can yield solid conclusions with a relatively small sample size (13). The experiment in the present study was terminated when seven crossover points (effective to ineffective) were achieved with 31 and 30 samples, respectively. Using the centered isotonic regression, the ED_50_ of RMZ was 0.263 mg/kg in Group B10 (10 μg/kg butorphanol) and 0.224 mg/kg in Group B15 (15 μg/kg butorphanol).

As a classic sedative in anesthesia for outpatient surgery, propofol has the strengths of fast onset time, profound sedative effect, and short duration. However, it produces significant respiratory and circulatory depression, increasing the risk for adverse events like hypoxemia and hypotension (15, 16). When used for procedural sedation, the sedative efficiency of RMZ was less than that of propofol as a novel IV sedative drug (17, 18). However, RMZ may be a safer sedative during anesthetic induction than propofol (9, 19). Many studies demonstrated that RMZ may reduce the incidence of hypotension, hypoxemia and injection-site pain compared to propofol, which is its most pronounced feature and advantage (7, 18, 20). Our findings revealed that the reduction of MAP in both groups was about 11%. This finding is consistent with Oka et al. (21). Only one patient in both groups had a maximum decrease in MAP (26.1%). However, no vasoactive medication is required, and the patient can recover relatively quickly. In either group, no patient experienced respiratory depression. The results indicate that RMZ has a little respiratory depressant effect. Therefore, our findings reveal that RMZ combined with butorphanol provided a good efficacy and safety profile in the sedation of painless artificial abortion.

This dual mechanism of action offers a balanced analgesic effect with reduced risk of respiratory depression compared to traditional opioid analgesics such as remifentanil and sufentanil. When administered intravenously, butorphanol has strong analgesic and sedative effects. Various studies have demonstrated that the incidence of adverse events of butorphanol is dose-dependent (22). Butorphanol is widely used in outpatient surgical anesthesia. However, there were differing views on the appropriate dose of butorphanol (3, 6), particularly for painless abortion. Butorphanol has a 3 to 5 min onset time, and RMZ should be administered 5 min after an IV bolus of butorphanol to maximize analgesic and sedative effects during a painless artificial abortion. We selected dosages of 10 μg/kg (Group B10) and 15 μg/kg (Group B15) of butorphanol based on our clinical experience, and aimed to compare the efficacy and safety of these two dosages to provide more precise guidance for clinical use. Our research indicated that the 10 μg/kg dosage of butorphanol appears to be more appropriate.

Butorphanol caused itching, somnolence, dizziness, nausea and vomiting among its adverse events (23). Although female patients exhibit heightened pain sensitivity (24), due to the analgesic effect of butorphanol, the visual analog scale (VAS) score for injection site pain and uterine contraction pain is still very low. Our findings revealed that the recovery time of Group B10 was faster than Group B15 (9.8 ± 2.3 vs. 12.5 ± 3.6 min, *p* < 0.05), which could be due to an increased incidence of somnolence and dizziness with an increase of butorphanol dosage, which significantly affects patient recovery time (22). However, the incidence of PONV was lower in Group B10 (3.2% vs. 16.7%) than in Group B15, but not statistically different (*p* = 0.104). This result could be attributed to a small sample size.

Our study has several strengths. This is one of the few studies conducted on first- trimester abortion patients exploring the clinical application of RMZ to meet procedural sedation requirements. The study aimed to determine the effective dose of RMZ in combination with two different dosages of butorphanol by applying the Dixon up-and-down method to calculate the ED_50_. The Dixon up-and-down method reduces excessive drug use, enabling more precise medication and reducing the potential risks of adverse reactions to achieve a balance between therapeutic effectiveness and safety. Additionally, the Dixon up-and-down method can help researchers determine the optimal dosage more quickly using a smaller sample size. This study obtained relatively accurate results using just 61 patients, thus saving the time and resources required for the research.

The present study has several limitations. First, we found that patients without a history of vaginal delivery had relatively stronger stimulation of cervical dilation than those with a history of vaginal delivery. Because the dose of the next patient depended on the response of the previous patient, thus individual differences may affect the accuracy of the final result. Second, our pre-trials demonstrated that consumption for RMZ use alone was very high in terms of inhibiting stimulus-to-response of cervical dilatation and was prone to hemodynamic instability. Therefore, the study design did not include the blank control group (RMZ use alone). Third, as the dosage is increased, the likelihood of adverse drug reactions increases. However, the two groups have no statistical difference in adverse events. We believe that the small sample size is the main reason, and if the sample size was larger, the ED_50_ value would be more accurate, and the 90% CI would be narrower (14).

In conclusion, the ED_50_ of RMZ combined with a 10 μg/kg or 15 μg/kg dose of butorphanol was 0.263 and 0.224 mg/kg during painless first trimester artificial abortion, respectively. However, RMZ combined with a 10 μg/kg dose of butorphanol seems to have a shorter recovery time.

## Data availability statement

The original contributions presented in the study are included in the article/supplementary material, further inquiries can be directed to the corresponding author.

## Ethics statement

The studies involving humans were approved by Ethics Committee of the Affiliated Shunde Hospital of Jinan University. The studies were conducted in accordance with the local legislation and institutional requirements. The participants provided their written informed consent to participate in this study. Written informed consent was obtained from the individual(s) for the publication of any potentially identifiable images or data included in this article.

## Author contributions

JC: Writing – original draft. XL: Data curation, Investigation, Writing – review & editing. ZH: Methodology, Project administration, Writing – review & editing. YZ: Data curation, Investigation, Writing – review & editing. YM: Methodology, Project administration, Writing – review & editing. ZZ: Conceptualization, Writing – review & editing.
